# Bidirectional relationship between sleep disturbances and pain in Japanese patients with chronic pain: findings from actigraphy and sleep diaries

**DOI:** 10.1007/s41105-025-00597-6

**Published:** 2025-07-14

**Authors:** Hidenori Harada, Ayaka Matsuo, Atsuo Yamashita, Mishiya Matsumoto

**Affiliations:** Department of Anesthesiology, Yamaguchi University Graduate School of Medicine, 1-1-1 Minami-Kogushi, Ube, Yamaguchi 755-8505 Japan

**Keywords:** Chronic pain, Sleep disturbance, Actigraphy, Sleep diary

## Abstract

This study aimed to evaluate the bidirectional relationship between sleep and pain in Japanese patients with chronic pain through a prospective analysis of the impact of pain on sleep and the impact of sleep on pain the following day, based on both objective and subjective measures. Sleep and pain parameters were recorded every day for a 7-day period in 36 patients with chronic pain. Objective sleep data were collected using an actigraph, and total sleep time, sleep onset latency, total wake time after sleep onset, and sleep efficiency were determined. Subjective ratings of sleep and pain intensity were obtained using sleep diaries and a pain scale. A mixed-effects model was used for data analysis, and the bidirectional relationship between pain and sleep was examined in each direction. Sleep efficiency measured by actigraphy was significantly higher on days when pain before sleep was less intense (p < 0.05), but pain intensity did not affect subjective sleep satisfaction. Sleep efficiency did not affect pain after sleep the next day, but pain after sleep was significantly less intense on days when sleep satisfaction was high (p < 0.0001). The results of this study suggest that the relationship between pain and sleep is bidirectional, but objective sleep data and subjective ratings of sleep were inconsistent. Improvement of perceived sleep quality may be necessary in patients who do not notice any change in their sleep quality despite achieving better sleep efficiency through treatment.

## Introduction

Chronic pain is defined as pain that persists or recurs for more than 3 months [1]. The prevalence of chronic pain ranges from 10 to 30% in Europe [2] and was reported to be 39.3% from a cross-sectional survey in Japan [3]. Chronic pain has wide-ranging effects, including dramatic reductions in quality of life (QOL) and high social and economic costs.

Many patients with chronic pain also experience sleep disturbances, with reported rates ranging from 24% to over 50% [4]. These sleep disturbances may worsen or prolong pain [5, 6] and require appropriate management.

Previous studies conducted outside Japan [7] have found that patients with chronic pain have shorter total sleep time, longer wake time after sleep onset, decreased sleep quality and satisfaction, longer sleep onset latency, and shorter deep sleep time. Sleep disturbances were historically considered a direct result of pain, but it is now widely recognized that pain and sleep have a bidirectional relationship. This relationship is considered to be a vicious cycle in which pain interferes with sleep and sleep disturbances increase sensitivity to pain [8].

Regarding the bidirectional relationship between pain and sleep disturbance, a study conducted over a 2- to 3-year period among community-dwelling older adults in Singapore and Japan found evidence of a bidirectional association in both groups [9]. However, when the analysis was restricted to individuals who had no pain at baseline, sleep disturbance was associated with the subsequent onset of pain among older adults in Singapore, but not among those in Japan [9]. The authors suggest that cultural differences may account for this discrepancy. In particular, it has been reported that Japanese individuals tend to be less accepting of pain behaviors compared with Westerners [10].

Previous studies conducted in non-Japanese populations have demonstrated bidirectional associations between pain and sleep disturbance at the day-to-day level among patients with chronic pain [11, 12]. However, to date, no such investigation has been conducted in the Japanese population. Given the potential cultural tendency toward stoicism in pain expression among Japanese individuals, as noted above, findings from studies outside Japan may not be fully generalizable to the Japanese population.

The aim of this study was to examine the daily-level association between pain and sleep disturbance in Japanese patients with chronic pain. Sleep can be evaluated with a degree of objectivity using several devices, and the importance of assessing sleep from both subjective and objective perspectives has been emphasized in the literature [13]. In this study, we employed actigraphy—a minimally intrusive device in daily life—to objectively assess sleep parameters.

## Materials and methods

### Participants

Eligible patients with chronic pain were recruited from outpatients attending the pain clinic at Yamaguchi University Hospital.

Inclusion criteria were as follows:

- Age ≥ 18 years
- Pain at the same site lasting ≥ 3 months
- Pain intensity of ≥ 3 on the numerical rating scale (NRS) at the time of consent
- Able to complete a medical questionnaire and sleep diary

Exclusion criteria were as follows:

- Cancer pain
- Psychiatric disorder with an obvious impact on pain and sleep (e.g., severe depression)
- Working night shifts
- Unable to wear an actigraph for long periods of time (e.g., due to working in an environment with strict hygiene standards or in construction)

No diseases or past treatments were set as exclusion criteria.

This study was approved by the Research Ethics Committee of Yamaguchi University Hospital.

Informed consent was obtained from all individual participants included in the study.

### Procedure

Participants completed a self-reported questionnaire about their pain, mental health (e.g., anxiety and depression), QOL, and sleep. After that, an actigraph (wGT3X-BT, ActiGraph, LLC; Pensacola, FL) was put on the non-dominant wrist of all participants. Participants were given written and verbal instructions explaining how to complete a sleep diary to self-evaluate their sleep for 8 consecutive days and how to use the actigraph. They were instructed to wear the actigraph at all times, except when bathing or showering, and to maintain their normal daily rhythm and sleeping/waking habits for the duration of the study. Participants were given a stamped mailing envelope to return the sleep diary and actigraph at the end of the study period. They were provided contact information for the institution conducting the study so that they could ask questions at any time during the study.

### Participant assessment

#### Self-reported questionnaires

At the time of enrollment, all participants completed a self-report questionnaire that included the following question items: basic clinical information (age, sex, diagnosis, disease duration); maximum, minimum, and average pain intensity within the past 24 h (NRS); Pain Catastrophizing Scale; Hospital Anxiety and Depression Scale; Athens Insomnia Scale; PainDETECT; and EuroQoL 5-Dimension.

The NRS is widely used to measure subjective sensations such as pain on a scale of 0 to 10. For pain intensity, participants self-rated their pain on an 11-point scale from 0 (no pain) to 10 (worst pain imaginable).

#### Daily subjective sleep assessment: sleep diary reports

**Fig. 1:**
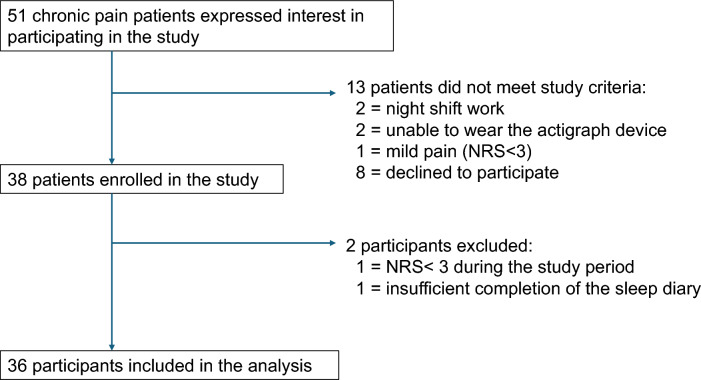
Patient flow diagram

The sleep diary included the following items, and participants were instructed to make entries before sleep and after waking (Fig. 1). Before sleep: activities done during the day (e.g., eating, working, going out); and pain before sleep (NRS). After waking: bedtime, wakeup time, quality of sleep (NRS), and pain after sleep (NRS).

Participants rated quality of sleep (NRS) on a scale from 0 (unsatisfactory) to 10 (satisfactory). Time in bed was defined as the time from bedtime to wakeup time. They recorded subjective ratings in the sleep diary for 8 days, from Day 1 to Day 8. However, to give participants time to get used to the actigraph, and to ensure that results reflected a weekly routine, only 7 days of data (from Day 2 to Day 8) were included in the analysis.

#### Daily objective sleep assessment: actigraphy

The following were evaluated as objective sleep data (Fig. 1): total sleep time, sleep onset latency, wake time after sleep onset, and sleep efficiency. Sleep was evaluated objectively using an actigraph (wGT3X-BT, ActiGraph, LLC) for 8 consecutive nights. An actigraph is a lightweight electronic device worn on the wrist and is used to measure physical activity and sleep with a three-axis accelerometer. Using specialized software (ActiLife 6 Data Analysis Software) and the Cole-Kripke scoring algorithm, we analyzed locomotor activity data in 60-s epochs to determine whether participants were asleep or awake.

Objective sleep data from the actigraph were combined with bedtime and wakeup time data from the sleep diary, and sleep onset latency (min) was calculated as the time from bedtime to sleep onset. Wake time after sleep onset (min) was calculated as the duration of wake epochs that occurred between sleep onset and the last wakeup. Total sleep time (min) was calculated as the total time in bed that was classified as sleep. Sleep efficiency was calculated using the following formula:

$${\mathrm{Sleep}}\;{\mathrm{efficiency}}\;\left( \% \right) = \left( {\text{Total sleep time}} \right)/\left( {\text{Time in bed}} \right) \times {1}00$$

#### Daily pain measurement

Participants recorded pain intensity ratings in their sleep diary twice a day—once before bedtime and once after waking—for 8 consecutive days. The change in pain after sleep (Δpain) was defined as the difference between pain from before sleep to after sleep:

$$\Delta {\mathrm{pain}} = \left( {\text{Pain after sleep}} \right){-}\left( {\text{Pain before sleep}} \right)$$

**Fig. 2:**
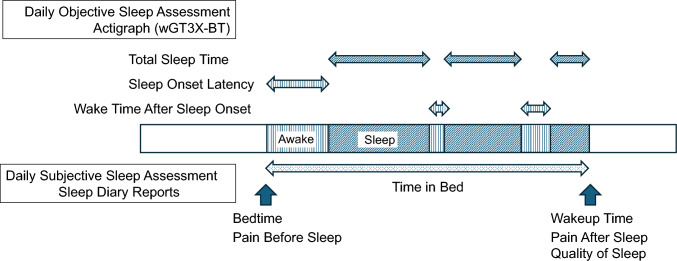
Sleep and pain assessments by actigraphy and sleep diaries. Daily objective sleep assessments were made using an actigraph (wGT3X-BT) for 8 days. Daily objective sleep assessments included total sleep time, sleep onset latency, and wake time after sleep onset. Daily subjective sleep assessments were self-recorded by participants in sleep diary reports. Daily subjective sleep assessments included bedtime, pain before sleep, wake time, quality of sleep, and pain after sleep

A positive Δpain indicates a higher pain intensity after sleep than before sleep, whereas a negative Δpain indicates a lower pain intensity after sleep (Fig. 2).

Relative pain before sleep was defined as pain intensity before sleep relative to the participant’s average pain intensity:

$$\left( {\text{Relative pain before sleep}} \right) = \left( {\text{Pain before sleep}} \right) - \left( {\text{Average pain}} \right)$$

A positive relative pain before sleep indicates higher than average pain, whereas a negative result indicates lower than average pain.

### Statistical analysis

To evaluate the impact of relative pain intensity before sleep on objective sleep data and subjective sleep assessments from that night, a mixed-effects model was used with objective sleep data (sleep efficiency, total sleep time, sleep onset latency, and wake time after sleep onset) and quality of sleep set as dependent variables, and relative pain before sleep as a fixed effect. To evaluate the impact of objective sleep data and subjective sleep assessments on changes in pain after sleep, a mixed-effects model was used with Δpain set as a dependent variable, and age, sex, objective sleep data (sleep efficiency, total sleep time, sleep onset latency, and wake time after sleep onset) and quality of sleep as fixed effects.

We accounted for inter-individual variability by including a patient identifier as a random effect in the mixed-effects model. This approach allowed us to capture overall trends while considering differences in baseline conditions and individual responses among patients. Specifically, the model incorporated repeated measurements for each patient, enabling us to appropriately handle the longitudinal data and account for intra-individual correlations.

JMP Pro 16 (SAS Institute Inc.; Cary, NC) was used for statistical analysis. Participant characteristics were summarized as means and standard deviations for continuous variables, and as frequencies and percentages for categorical variables. All statistical tests were two-sided, with a significance level of 0.05.

## Results

### Patient demographic and clinical characteristics

A total of 51 patients with chronic pain expressed interest in participating in the study. Fifteen patients were excluded, and 38 patients were enrolled. All participants completed their sleep diary and wore the actigraph for 8 days. Data from 2 participants were excluded: 1 participant provided insufficient sleep diary data, and 1 participant reported pain intensity below 3 throughout the entire study period. Thus, data from 36 participants were available and included in the analysis. One participant did not wear their actigraph during 1 night of the 7-day analysis period from Days 2 to 8. Consequently, 251 days of data for 36 participants were included in the analysis, with all participants wearing their actigraph for at least 85% of the study period.

Table 1 shows the clinical characteristics of participants. Most participants had relatively intense pain in the 24 h prior to providing informed consent, with a maximum NRS of 7.3, minimum of 3.5, and mean of 5.8. The mean Pain Catastrophizing Scale score was 28.9, below the cutoff of 30. On the Hospital Anxiety and Depression Scale, the mean scores for Anxiety and Depression were 6.6 and 6.0, respectively, which were both below the cutoff of 8 that indicates a possible mental health condition. These results demonstrate that most participants did not have major psychological or social problems.

**Table 1:** Demographic and clinical characteristics of patients

Variables	N = 36
Age (years)	59.2 (13.6)
Max pain (0–10)	7.3 (2.1)
Min pain (0–10)	3.5 (2.5)
Average pain (0–10)	5.8 (1.8)
PCS (0–42)	28.9 (10.0)
HADS-anxiety (0–14)	6.6 (3.9)
HADS-depression (0–14)	6.0 (3.8)
EQ-5D (0–1)	0.650 (0.159)
AIS (0–24)	6.4 (4.3)
PainDETECT (0–38)	13.7 (5.5)
	*n (%)*
Sex, female	25 (69)
Disease breakdown	MSK pain: 18, FM: 10, PHN: 2, PP: 2, Headache/facial pain: 2, MS: 1, CRPS: 1
*Medication*	
Sleep medication, yes	15 (41)
Opioid, yes	18 (50)
Gabapentinoid, yes	22 (61)
Antidepressant, yes	20 (56)

Data are expressed as the mean (standard deviation)

*PCS*  pain catastrophizing Scale, *HADS*  Hospital Anxiety and Depression Scale, *EQ5D*  EuroQol 5-Dimension, *AIS*  Athens Insomnia Scale, *MSK*  musculoskeletal, *FM*  fibromyalgia, *PHN*  postherpetic neuralgia, *PP*  postoperative pain, *MS*  multiple sclerosis, *CRPS*  complex regional pain syndrome

On the Japanese version of the EuroQol 5-Dimension, a measure of patient QOL, the mean score was 0.650, indicating a slightly impaired QOL. On PainDETECT, which shows characteristics of pain, the mean score was 13.7, indicating that many participants experienced a mix of neuropathic and nociceptive pain. The mean score on the Athens Insomnia Scale was 6.4, indicating a high number of participants with suspected insomnia.

### Relationship between pain and sleep

#### Impact of pain before sleep on sleep

Table 2 shows the impact of relative pain intensity before sleep on objective sleep data and subjective sleep assessments. Mixed-model analysis showed that sleep efficiency, an objective measure of sleep, improved when pain intensity before sleep was lower than average. Specifically, a 1-point decrease in relative pain before sleep was associated with a 1.33% increase in sleep efficiency (p < 0.05). However, there were no significant differences in total sleep time, sleep onset latency, wake time after sleep onset, or subjective quality of sleep.

**Table 2:** Results from the mixed-effects model with multiple dependent variables

Dependent variable	Fixed effect	Estimate	SE	t-value	p-value	95% CI
Sleep efficiency	Relative-NP	− 1.33	0.64	− 2.07	< 0.05*	[− 2.60, − 0.064]
TST	Relative-NP	− 7.92	5.35	− 1.48	0.14	[− 18.5, 2.62]
SOL	Relative-NP	1.15	0.85	1.35	0.18	[− 0.54, 2.84]
WASO	Relative-NP	4.99	3.13	1.59	0.11	[− 1.18, 11.2]
QOS	Relative-NP	− 0.11	0.10	− 1.08	0.28	[− 0.31, 0.09]

*SE*  standard error, *CI*  confidence interval, *TST*  total sleeping time, *SOL* sleep onset latency, *WASO*  wake time after sleep onset, *QOS*  quality of sleep, *NP*  night pain

#### Impact of sleep on pain after sleep

Table 3 shows the impact of objective sleep data and subjective sleep assessments on pain after sleep. Mixed-model analysis showed that a higher subjective quality of sleep was associated with a greater improvement in pain after sleep. Specifically, a 1-point improvement in quality of sleep was associated with 0.24 points of improvement in Δpain (p < 0.0001). However, there were no significant differences in age, sex, and objective sleep data (sleep efficiency, total sleep time, sleep onset latency, and wake time after sleep onset).

**Table 3:** Results from the mixed-effects model predicting Δpain

Fixed effect	Estimate	SE	t-value	p-value	95% CI
Intercept	0.51	2.76	0.19	0.85	[− 4.91, 5.95]
Age	− 0.03	0.022	− 1.71	0.096	[− 0.081, 0.007]
Sex (female)	0.49	0.28	1.77	0.086	[− 0.074, 1.1]
Sleep efficiency	0.03	0.029	1.01	0.31	[− 0.028, 0.088]
TST	0.00024	0.0015	0.16	0.87	[− 0.0028, 0.0032]
SOL	0.0075	0.0077	0.97	0.33	[− 0.0078, 0.023]
WASO	0.0037	0.0051	0.73	0.47	[− 0.0062, 0.13]
QOS	-0.24	0.051	− 4.62	< 0.0001*	[− 0.34, − 0.13]

*SE* standard error, *CI* confidence interval, *TST*  total sleep time, *SOL*  sleep onset latency, *WASO* wake time after sleep onset, *QOS* quality of sleep

## Discussion

In this study, we evaluated the bidirectional relationship between pain and sleep at a daily level in Japanese patients with chronic pain, and we found that pain and sleep may have interactive effects. Specifically, we confirmed that lower relative pain at night before sleeping is associated with higher sleep efficiency, and higher sleep satisfaction is associated with lower pain intensity after waking.

Few studies to date have investigated the bidirectional relationship between pain and sleep in patients with chronic pain. Tang et al. [11] investigated sleep, mood, and pain in 119 British patients with chronic pain using an electronic diary and an actigraph, specifically the Actiwatch-Insomnia model (Cambridge Neurotechnology Ltd., Cambridge, UK). They found that worsening pain before sleep decreased sleep efficiency and sleep quality based on sleep diary records, but did not affect objective sleep data based on actigraphy. In addition, decreased sleep quality and sleep efficiency based on sleep diary records was associated with higher pain intensity the next morning, but objective sleep data based on actigraphy did not affect pain intensity the next morning. Alsaadi et al. [12] evaluated sleep and pain in 80 Australian patients with chronic low back pain using sleep diaries and an actigraph, specifically the SenseWear Pro3 Armband (BodyMedia, Pittsburgh, PA). Pain before sleep was associated with lower sleep quality and sleep efficiency based on sleep diary records, as well as lower sleep efficiency and increased total wake time after sleep onset based on actigraphy. In addition, lower sleep efficiency and lower sleep quality based on sleep diary records, as well as lower sleep efficiency and longer total wake time based on actigraphy, were associated with higher pain intensity the next morning.

These studies differ in that Tang et al. found that only subjective sleep assessments had a bidirectional relationship with pain, whereas Alsaadi et al. found that both objective sleep data and subjective sleep assessments had a bidirectional relationship with pain. This difference can most likely be attributed to the model of actigraph used in each study. The actigraph used by Tang et al. is a basic model that uses body movements to determine sleep status, whereas the actigraph used by Alsaadi et al. is a multifunctional model that more accurately determines sleep status by simultaneously collecting physiological data such as skin temperature, electrodermal activity, and heat flow in addition to monitoring body movements. This additional physiological data likely enabled more accurate definition of the boundary between waking and sleep, and improved the accuracy of objective sleep data.

The actigraph used in this study was a basic actigraph that determines the wearer’s sleep state from body movements, similarly to the device used by Tang et al. Some people, including patients with chronic pain, are often stationary but awake for long periods of time from bedtime to falling asleep or during the night, and studies have noted that actigraphs may incorrectly classify these stationary periods as “sleep,” leading to overestimation of sleep efficiency in such groups [14, 15]. Although multifunctional actigraphs are highly accurate, we believed that patients with chronic pain would find these devices uncomfortable to wear for many days due to their large size and how they are worn on the arm. Therefore, we selected a small actigraph worn on the wrist like a watch to impose a smaller burden on participants.

In the present study involving Japanese patients with chronic pain, we found that pain before sleep significantly influenced objective sleep assessments of sleep efficiency but did not have a significant effect on subjective sleep data. These findings contrast with those of Tang et al. [11], who reported different patterns in Western populations, suggesting that cultural factors may play a role. Japanese individuals are reported to adopt a self-restrained and introspective attitude toward pain, often exhibiting a form of “stoicism” in which outward expressions of pain behavior are minimized [10]. Such cultural tendencies may extend to the evaluation of sleep as well, whereby individuals may refrain from reporting sleep disturbances even when they are present. Therefore, in clinical settings, it is important to ask specific and objective questions such as “How many times did you wake up during the night?” or “How long did it take you to fall asleep?” rather than to rely solely on general subjective inquiries such as “Did you sleep well?”, as the latter may fail to capture the actual presence of sleep disturbances.

In both our study and the study by Tang et al., objective sleep data and subjective sleep assessments had an inconsistent impact on pain the next day. This phenomenon where a patient’s subjective perception of their sleep is inconsistent with objective sleep data is called “sleep misperception,” and patients with chronic pain are considered particularly prone to sleep misperception because they tend to judge sleep quality based on their physical symptoms upon waking [16, 17]. This suggests that participants’ subjective evaluation of sleep may have been influenced by the intensity of pain, as they assessed both parameters simultaneously upon waking. It also highlights the importance of carefully distinguishing between subjective and objective assessments when evaluating sleep quality.

## Limitations

Our study has the following limitations. First, this study did not include a comparison with healthy controls, so it is unclear whether our findings are specific to patients with chronic pain. Second, the sample size may be small. However, using a mixed-effects model, we were able to show the unique findings that sleep efficiency measured by actigraphy was significantly higher on days when pain before sleep was less intense and that pain intensity did not affect subjective sleep satisfaction, while subjective sleep satisfaction affected pain intensity after sleep. Third, because of cultural influences, Japanese individuals tend to under-report subjective symptoms such as pain. Consequently, relying solely on the NRS in this study may have introduced response bias, thereby hindering an accurate elucidation of the true association between pain and sleep disturbance. Incorporating daily behavioral or activity records possibly would have enabled a more accurate assessment of pain. The single-center design is also a limitation. However, strong bias in the study population is unlikely because psychological indicators such as depression, anxiety, and catastrophizing were within normal ranges. Further research at other institutions will enable generalization of the results.

## Conclusion

This study evaluated the relationship between pain and sleep in Japanese patients with chronic pain using actigraphy and sleep diaries, and confirmed that lower pain intensity before sleep was associated with better sleep efficiency, but did not affect sleep satisfaction. Higher sleep satisfaction, but not sleep efficiency, was associated with less intense pain the following day. This suggests a bidirectional relationship between pain and sleep, but objective sleep data and subjective sleep assessments were inconsistent. Japanese individuals may be culturally inclined to refrain from expressing not only pain but also sleep problems. Therefore, it may be necessary to assess their actual condition through specific and objective questions during clinical interviews in order to improve perceived sleep quality in patients who do not notice any change in their sleep quality despite achieving better sleep efficiency through treatment.
